# *Helicobacter hepaticus* CdtB Triggers Colonic Mucosal Barrier Disruption in Mice via Epithelial Tight Junction Impairment Mediated by MLCK/pMLC2 Signaling Pathway

**DOI:** 10.3390/vetsci12020174

**Published:** 2025-02-14

**Authors:** Tao Wang, Xiao Meng, Miao Qian, Shanhao Jin, Ruoyu Bao, Liqi Zhu, Quan Zhang

**Affiliations:** 1 Institute of Comparative Medicine, College of Veterinary Medicine, Yangzhou University, Yangzhou 225009, China; 2 Co-Innovation Center for Prevention and Control of Important Animal Infectious Diseases and Zoonoses, Yangzhou University, Yangzhou 225009, China; 3 Jiangsu Transgenic Animal Pharmaceutical Engineering Research Center, Yangzhou University, Yangzhou 225009, China

**Keywords:** *Helicobacter hepaticus*, CdtB, colonic mucosal barrier, tight junction, MLCK, pMLC2

## Abstract

**Background:** *Helicobacter hepaticus* (*H. hepaticus*) has been demonstrated to have clinical relevance to the development of colitis in rodents. *H. hepaticus* produces cytolethal distending toxins (CDTs), which are identified as the most important virulence factors to the pathogenicity of CDT-producing bacteria in animals. However, the precise relationship between CDTs of *H. hepaticus* and intestinal barrier dysfunction remains unclear. The objective of the present study was to ascertain the impact of CdtB, the active subunit of CDTs, on the colonic mucosal barrier during *H. hepaticus* infection. **Materials and Methods**: We investigated the infection of male BALB/c mice, intestinal organoids, and IEC-6 cell monolayers by *H. hepaticus* or CdtB-deficient *H. hepaticus* (ΔCdtB). A comprehensive histopathological examination was conducted, encompassing the assessment of *H. hepaticus* colonization, the levels of mRNA expression for inflammatory cytokines, the expression levels of tight junction proteins, and the related signaling pathways. **Results**: The results demonstrate that the presence of ΔCdtB led to a mitigation of the symptoms associated with *H. hepaticus*-induced colitis, as evidenced by colon length shortening and the colon histological inflammation score. In addition, the levels of pro-inflammatory cytokines were reduced in the ΔCdtB group. Moreover, a downward trend was observed in the phosphorylation levels of STAT3 and nuclear factor-κB (p65). In vitro, the presence of *H. hepaticus* resulted in a reduction in the expression of tight junction (TJ) markers (ZO-1 and occludin) and an impairment of the F-actin structure in either the intestinal epithelium or intestinal organoids. However, these effects were reversed by CdtB deletion. Concurrently, both ROS levels and apoptosis levels were found to be significantly reduced in cells treated with the ΔCdtB strain. Mechanistically, myosin light chain kinase (MLCK) activation was observed in the *H. hepaticus*-infected group in vivo, whereas the MLCK inhibitor ML-7 was found to reverse the CdtB-induced alterations in TJ proteins in IEC6 cells. **Conclusions**: The collective findings demonstrate that CdtB plays a pivotal role in the *H. hepaticus*-induced colonic mucosal barrier. This is achieved through the regulation of TJs via the MLCK/pMLC2 signaling pathway, which is linked to elevations in oxidative stress and inflammation within intestinal epithelial cells.

## Simple Summary

Helicobacter hepaticus (*H. hepaticus*) has been demonstrated to have clinical relevance to the development of colitis in rodents. The aim of the present study was to ascertain the impact of CdtB, the active subunit of CDT, on the colonic mucosal barrier during *H. hepaticus* infection. The results demonstrate that the presence of ΔCdtB led to a mitigation of the symptoms associated with *H. hepaticus*-induced colitis. In vitro, CdtB mutation reversed the reduction in tight junction (TJ) marker (ZO-1 and occludin) expression and the impairment of the F-actin structure induced by *H. hepaticus* in either IEC6 cells or intestinal organoids. ROS levels were found to be significantly reduced in cells treated with the ΔCdtB strain. Furthermore, myosin light chain kinase (MLCK) activation was observed in the *H. hepaticus*-infected group in vivo, whereas the MLCK inhibitor ML-7 was found to reverse the CdtB-induced alterations in TJ proteins in IEC6 cells. CdtB plays a pivotal role in the *H. hepaticus*-induced colonic mucosal barrier. This is achieved through the regulation of TJs via the MLCK/pMLC2 signaling pathway, which is linked to elevations in oxidative stress and inflammation within intestinal epithelial cells.

## 1. Introduction

Members of the genus *Helicobacter* have been reported in a wide range of animals, and they have been identified to infect numerous sites, including the stomach, small intestine, large intestine, liver, and bile duct, and they have even been reported in the hearts of chickens and in the milk of sheep and cows [1,2]. *H. hepaticus*, a close relative of *H. pylori* and *C. jejuni*, is a member of the enterohepatic *Helicobacter* species (EHS) [3]. It colonizes the colons, ceca, and hepatobiliary tracts of rodents [4,5,6] and has been reported to cause colitis and hepatitis in susceptible mouse strains in past decades [7,8,9,10]. Pathobionts are defined as members of the gut microbiota that possess the capacity to induce diseases when intestinal homeostasis is disrupted. The hypothesis that these organisms play a significant role in the etiology of enteritis and inflammatory bowel disease (IBD) is currently under investigation. IBD is a group of chronic inflammatory disorders characterized by an aberrant immune response to microbiota. Gut microbiota comprise both beneficial and harmful microorganisms, and enterohepatic *Helicobacter* spp. infection is considered a naturally acquired condition [11]. Intestinal bacterial dysbiosis and *H. hepaticus* infection exert a synergistic effect on the development of IBD and neoplastic progression. The recent detection of *H. hepaticus* in bile samples from patients with pancreatic and biliary tract cancers indicates a potential role for this organism in the progression of gastrointestinal diseases in humans [12,13]. Therefore, a deeper comprehension of the underlying mechanism of *H. hepaticus*-induced colonic mucosal barrier damage is crucial for the advancement of human and animal health.

In a manner analogous to other pathogenic bacteria, *H. hepaticus* produces and secretes cytolethal distending toxin (CDT), which is a critical factor in the development of colitis. CDT is classified as an AB_2_ toxin, a category of toxins produced by Gram-negative bacteria [3,14], and consists of CdtA, CdtB, and CdtC [15,16]. The genome of *H. hepaticus* consists of 1,799,146 base pairs (bps), and the CDT gene is located from 1,395,180 bp to 1,398,116 bp. The length of CDT coding DNA sequence is 2070 bp, consisting of CdtA (696 bp), CdtB (822 bp), and CdtC (552 bp) in order. The virulence of CDT is dependent on CdtB, which has DNase activity [16]. As CdtB contributes to the pathogenicity of CDT-producing bacteria in vivo, it is of great significance to explore the pathogenic mechanism of CdtB to ensure the health of humans and animals. The biological functions of CdtB have been documented to include G2/M cell cycle arrest, STAT3 activation, DNA double-strand breaks (DSBs), and apoptosis in intestinal epithelial cells (IECs), which can lead to the development of epithelial dysplasia [16,17,18].

The surface of the intestinal mucosa is lined with epithelial cells that provide an essential barrier to the diffusion of pathogens, toxins, and allergens from the lumen to the mucosal tissues [19]. Tight junctions represent a crucial element of the intestinal barrier, comprising the assembly of multiple proteins situated at the apical aspect of the outer membrane of epithelial cells, including ZO-1, occludin, and claudin. The aberrant expression and distribution of tight junction proteins in intestinal tissue result in increased intestinal permeability and dysfunction of the intestinal epithelial barrier [20,21]. Our previous work has demonstrated that *H. hepaticus* CdtB is capable of causing disruption to the intestinal mucosa in IL10^−/−^ mice [17] and can induce intestinal epithelial cell death via the mitochondrial apoptotic pathway in vitro [22]. However, it remains unknown whether *H. hepaticus* CdtB affects the intestinal barrier and the pathogenesis of colitis.

The objective of this study was to investigate the effect of *H. hepaticus* CdtB on the intestinal barrier. The data presented herein suggest a potential effect of CdtB on colitis, which is associated with tight junction dysfunction and cytoskeletal disorders. Our study can provide better insights regarding pathogenic mechanisms and treatment strategies for EHS-triggered enteritis.

## 2. Materials and Methods

### 2.1. Animals

Male BALB/c mice (6∼8 weeks old) were purchased from the Comparative Medicine Center of Yangzhou University (male BALB/c mice are more sensitive to *H. hepaticus*). The animal experiments were conducted in accordance with the project license [SYXK (Su) 2022-0044] approved by Jiangsu Provincial Science. The ethics of the use of laboratory animals (202203022) was approved by the Institutional Animal Care and Use Committee (IACUC) of Yangzhou University. The mice utilized in this study were free of *Helicobacter* species, as determined by PCR. The mice were randomly divided into three groups, with 30 mice in each group, as the euthanasia was performed in two batches. The *H. hepaticus* and *H. hepaticus* ΔCdtB groups were administered the microorganism (1 × 10^8^ CFU) in 200 μL PBS solution by gavage, while the control group was given 200 μL PBS by gavage every 2 days for a week. Fecal samples were collected on a weekly basis and subjected to PCR analysis to ascertain the presence of infection. Mice from each group were euthanized at the 4th and 9th weeks post-infection (WPI), respectively. Colonic tissues were collected for subsequent experiments.

### 2.2. Intestinal Organoid Models

The methodology for separating colon organoids is based on the reports of Hiroyuki Miyoshi et al. [23]. In brief, the mice were fasted and provided with water containing a mixture of penicillin, streptomycin, and gentamicin one day prior to sacrifice. Subsequently, the colon samples were collected, longitudinally dissected, and washed repeatedly in PBS containing penicillin, streptomycin, and amphotericin B. The samples were then gently shaken with digestion solution (20 mM EDTA in Ca^2+^/Mg^2+^-free PBS) at 37 °C in order to dissociate the epithelial layer. Subsequently, low-speed centrifugation was employed to separate the supernatant, which contains impurities and individual cells. The crypts were present in the precipitate and were diluted with the organoid culture medium to a concentration of 1 × 10⁴ crypts/mL. The crypt suspension was combined with Matrigel in a 1:1 ratio and inoculated into a 24-well plate at a volume of 50 μL per well. The organoid culture medium was formulated with 1 × N_2_, 1 × B27, 1% GlutaMAX, 12.5 mM NAC, 50 ng/mL EGF, 15% L-WRN medium, 10 μM Y27632, and 10 μM SB-431542 in Advance DMEM/F12.

### 2.3. Cell Culture and Treatment

IEC-6 cells (ATCC^®^ CRL-1592™) were cultivated in Dulbecco’s modified Eagle’s medium (DMEM, ATCC^®^ 30-2002™), with the addition of 10% fetal bovine serum (FBS, Gibco), within a humidified atmosphere containing 95% air and 5% CO_2_ at a temperature of 37 °C. The cells were seeded in 24-well plates and subsequently exposed to *H. hepaticus* and recombinant *H. hepaticus* CdtB protein once confluent monolayers had formed.

### 2.4. Bacterial Culture

In our previous study, a mutant strain of *H. hepaticus* (*H. hepaticus* ΔCdtB) that lacks the CdtB gene was constructed. The *H. hepaticus* 3B1 (ATCC 51449) and *H. hepaticus* ΔCdtB strains were cultured on Brucella agar plates supplemented with 5% sheep blood and antibiotics (amphotericin B, vancomycin, and cefoperazone) for 4–5 days under microaerobic conditions (85% N_2_, 10% CO_2_, and 5% O_2_) at 37 °C. *H. hepaticus* was harvested in PBS and employed for research purposes until the optical density (OD) 600 reading of the bacterial solution reached 1.

### 2.5. DNA Extraction and PCR Analysis

The colons were collected in the same position, and the tissue and bacterial DNA were extracted according to the manufacturer’s instructions using the Bacteria DNA Extraction Kit (Vazyme, Nanjing, China). The quantification of *H. hepaticus* in the colon was performed using quantitative polymerase chain reaction (qPCR) with the Applied Biosystems Step One Real-Time PCR System (ABI) and Universal SYBR Green Master (ABI). The level of colonization by *H. hepaticus* was estimated by normalization to micrograms of mouse chromosomal DNA.

### 2.6. Histopathology

During the necropsy, the colon tissues of each animal were fixed in 4% paraformaldehyde for a period of 24 h. Thereafter, the proximal, middle, and distal parts were embedded in paraffin for microscopic examination. Tissue sections (5 μm) were subjected to staining with hematoxylin–eosin (H&E) and alcian blue. The histological scores were evaluated in accordance with the previously described methodology, taking into account the extent of the inflammatory infiltrate in the mucosa, submucosa, and muscularis/serosa; the severity of epithelial damage; the degree of crypt atrophy; and the presence of submucosal hyperplasia.

### 2.7. Immunohistochemistry Analysis

Prior to immunostaining, the paraffin-embedded sections were dewaxed and rehydrated in an alcohol series. Subsequently, the sections were treated with 3% hydrogen peroxide, after which antigen repair was performed with EDTA–citrate solution. Following this, bovine serum albumin was incubated with the sections. Subsequently, the sections were incubated overnight at 4 °C with diluted primary antibodies, including ZO-1 (sc-33725, 1:1000, Santa Cruz Biotechnology, Santa Cruz, CA, USA) and occludin (1:1000, Cambridge, MA, USA), for 16 h at 4 °C. The sections were developed using 3,3′-diaminobenzidine tetrahydrochloride (DAB) (VECTOR Laboratories, Burlingame, CA, USA). The nuclei were then counterstained with H&E for 1 min. The sections were then observed under a light microscope.

### 2.8. Cell Viability Assay

Cells were seeded into 96-well plates at a density of 1 × 10^4^ cells per well and incubated for 24 h. C-terminal octapeptide of cholecystokinin (CCK-8) assay (Beyotime, Shanghai, China) was used to assess cell viability. The OD at 450 nm was detected according to instructions via Synergy H1 Microplate Reader.

### 2.9. ROS Assessment

After treated with indicated conditions, cells were harvested and stained with DCFH-DA (Beyotime, Shanghai, China) according to the manufacturer’s instructions. The ROS were then determined by flow cytometry (excitation at 488 nm and emission at 525 nm).

### 2.10. Apoptosis Assessment

Following the completion of the treatment regimen, the cells were harvested and subjected to staining with Annexin V-FITC and PI in accordance with the instructions provided by the manufacturer (Solarbio, Beijing, China). Subsequently, the percentage of apoptotic cells was determined by flow cytometry.

### 2.11. Real-Time RT-PCR

Total RNA from the IEC-6 cells and colon tissues were extracted using Trizol reagent (Vazyme, Nanjing, China). cDNA synthesis was performed using PrimeScript™ RT reagent kit with gDNA Eraser (TaKaRa, Dalian, China). The mRNA levels of the inflammatory factors and the housekeeping gene *GAPDH* were detected using Universal qPCR SYBR Green Master Mix (Vazyme, Nanjing, China) performed on Step One Real-Time PCR System. Relative expression of target gene mRNA was calculated using the 2^−ΔΔCt^ method. Primers (5′-3″) used in this study are listed as follows:

*ZO-1* TTGCCACACTGTGACCCTA (forward), GGGGCATGCTCACTAACCTT (reverse); *occludin* GACCTTGTCCGTGGATGACTTCAG (forward), ATCAGCAGCAGCCATGTACTCTTC (reverse); *IL-6* GATACCACTCCCAACAGAC (forward), CTTTTCTCATTTCCACGAT (reverse); *TNF-α* TCCCAGAAAAGCAAGCAACC (forward), TAGACAGAAGAGCGTGGTGG (reverse); *IL-1β* GTGGTGTGTGACGTTCCCATTA (forward), CCGACAGCACGAGGCTTT (reverse); *iNOS* GTTGAAGACTGAGACTCTGG (forward), GACTAGGCTACTCCGTGGA (reverse); *GAPDH* AGGTCGGTGTGAACGGATTTG (forward), TGTAGACCATGTAGTTGAGGTCA (reverse).

### 2.12. Western Blot Assay

Samples from tissues or cells were collected, and the proteins were extracted from using cold 1 × RIPA buffer (Solarbio, China) and phenylmethylsulfonyl fluoride (PMSF) (1:1000). Protein concentration was measured with a bicinchoninic acid (BCA) protein assay kit (Solarbio) and then separated by 8–15% SDS-PAGE and transferred to PVDF membranes. The membrane was blocked with 5% skimmed milk for 1 h and incubated with the primary antibodies ZO-1 (sc-33725, 1:1000, Santa Cruz Biotechnology), occludin (1:1000, Abcam, Cambridge, UK), MLCK (sc-365352, 1:1000, Santa Cruz Biotechnology), p-MLC antibody (1:1000, Cell Signaling Technology, Danvers, MA, USA), MLC antibody (1:1000, Cell Signaling Technology, USA), and GAPDH (1:1000, Cell Signaling Technology, USA) and then incubated with secondary antibodies (1:5000, AB clonal Technology, Wuhan, China) labeled with horseradish peroxidase (HRP) at room temperature for 1 h. The bands were visualized using Amer sham ECL Select Western blotting detection reagent (Millipore, Miamisburg, OH, USA).

### 2.13. Fluorescence Assay

The colon tissues or cells were fixed with 4% paraformaldehyde for 15 min, after which they were blocked with 5% BSA (Beyotime, Shanghai, China). The samples were incubated with the indicated primary antibody and secondary antibodies labeled with FITC or rhodamine, respectively. The nuclei were stained with 4′,6-diamidino-2-phenylindole (DAPI; Beyotime, Shanghai, China). The cytoskeleton was stained with phalloidin (Invitrogen, Waltham, MA, USA). The samples were observed using a confocal microscope (Leica, Wetzlar, Germany). Excitation was achieved at 488 nm, with emission occurring at 530 nm.

### 2.14. Statistical Analysis

All studies were performed as three independent experiments. The data are expressed as the mean ± SD. Significant variance between groups was determined using one-way ANOVA following by Student’s *t*-test. *p* < 0.05 was considered statistically significant. Statistical analyses were performed by using Prism 7 software (GraphPad Software, Inc., La Jolla, CA, USA) and SPSS version 18.0 (SPSS, Inc., Chicago, IL, USA).

## 3. Results

### 3.1. CdtB Deficiency Alleviates H. hepaticus-Triggered Intestinal Inflammation

**Figure 1:**
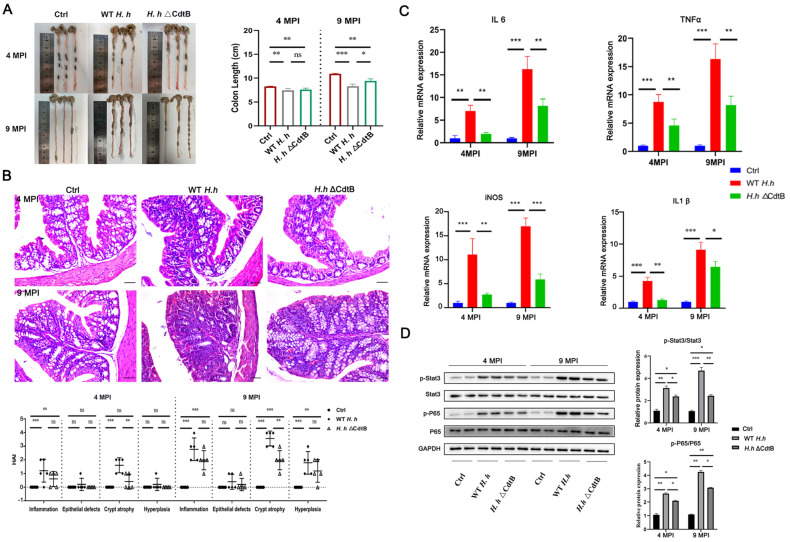
The etiology of colitis induced by *H. hepaticus* is dependent on the presence of CdtB. The mice were treated with *H. hepaticus*, with or without a CdtB mutation, via gavage for either four or nine months. (**A**) Representative colons were photographed, and the lengths were subsequently analyzed. (**B**) Representative images of colon tissue stained with H&E. The histology score was evaluated in accordance with the following criteria: inflammation, damage to the epithelial tissue, atrophy of the crypts, and hyperplasia. The scale bar represents a length of 50 μm. (**C**) The relative mRNA expression levels of IL-6, TNF-α, iNOS, and IL-1β in the mouse colon tissues were quantified by qPCR. (**D**) The relative expression levels of Stat3, p-Stat3, P65, and p-P65 proteins were quantified by a Western blot analysis. GAPDH was employed as a loading control. * *p* < 0.05, ** *p* < 0.01, and *** *p* < 0.001. ns: not significant.

The colonization of *H. hepaticus* in the colons of mice was detected at 4 or 9 MPI by using qPCR. As qPCR data lack an RNA integrity assessment and do not report primer efficiencies, colonization was identified by using an indirect immunofluorescence assay. The polyclonal *H. hepaticus* antibody, prepared in our previous study, was used to detect the *H. hepaticus*-specific antigen in the colons of both the WT and ΔCdtB-infected groups. As illustrated in Figure S1A,B, there is no difference in bacterial colonization between the two groups at either 4 MPI or 9 MPI (*p* > 0.05). Moreover, the colons of mice infected with WT *H. hepaticus* were significantly shorter than those of the control group, which was alleviated by the absence of CdtB (Figure 1A). Next, the colonic histopathology was evaluated. As illustrated in Figure 1B, there was a notable increase in inflammatory cell infiltration within the colons and altered crypt morphology in the WT *H. hepaticus*-infected mice at 4 MPI in comparison to the control mice. At 9 MPI, a more pronounced infiltration of inflammatory cells was evident, accompanied by the formation of lymphocyte aggregates and hyperplasia, within the submucosa and mucosal epithelium. Additionally, a markedly altered crypt morphology, characterized by either atrophy or dilatation, was observed. However, all of these pathological changes were observed to be impaired in mice infected with the ΔCdtB strain. Furthermore, the transcription of inflammatory cytokines, including IL-6, IL-1β, TNF-α, and iNOS, driven by *H. hepaticus*, was inhibited by CdtB deletion at both 4 MPI and 9 MPI (Figure 1C,D). Similarly, the elevated levels of p65 and STAT3 phosphorylation were also inhibited by the CdtB deficiency. In conclusion, these findings suggest that CdtB plays a pivotal role in *H. hepaticus*-induced intestinal inflammation, which involves the STAT3 and NF-κB signaling pathways.

### 3.2. CdtB Contributes to H. hepaticus-Induced Intestinal Barrier Damage

**Figure 2:**
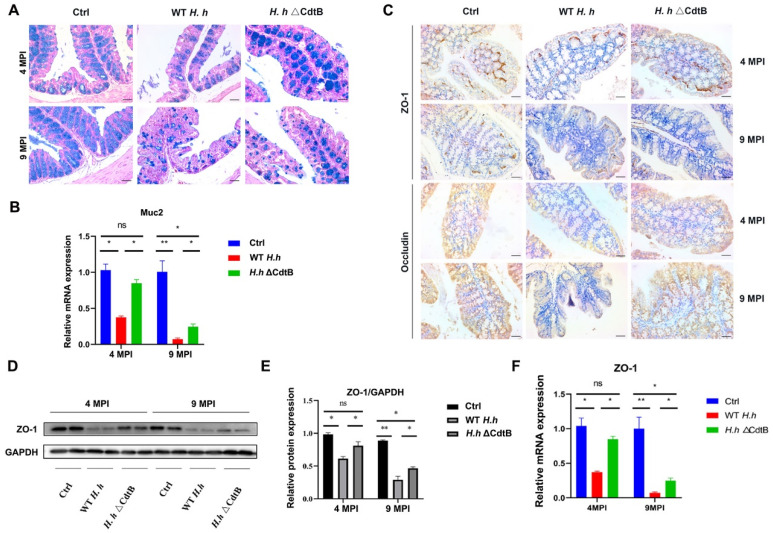
CdtB deficiency alleviates *H. hepaticus*-induced colonic mucosal barrier. Mice were treated with WT *H. hepaticus* or ΔCdtB strain by gavage for 4 and 9 months. (**A**) Representative images of alcian blue-stained colon tissues. (**B**) The relative mRNA expression levels of Muc2 in the mouse colon tissues were tested by q-PCR. (**C**) Representative immunohistochemistry of ZO-1 and occludin staining for colon sections are shown. Scale bar: 50 μm. (**D**,**E**) The relative expression levels of ZO-1 protein were measured by Western blotting. GAPDH was used as a loading control. (**F**) The relative mRNA expression level of ZO-1 was tested by q-PCR. * *p* < 0.05; ** *p* < 0.01. ns not significant.

To investigate whether CdtB is involved in *H. hepaticus*-induced mucin damage, colon sections were stained with alcian blue. As shown in Figure 2A, mice infected with WT *H. hepaticus* showed a significant decrease in the area of acidic mucin in the blue region compared to the control mice, which is reversed by CdtB deletion. The mucus barrier mucin2 (Muc2) acts as the first barrier preventing direct contact between intestinal bacteria and colonic epithelial cells [24]. Similarly, the reduction in Muc2 transcription resulting from *H. hepaticus* infection was also prevented by the CdtB mutation (Figure 2B). Moreover, a reduction in zonula ZO-1 and claudin-1 protein distribution was evident in the intestinal epithelium of mice treated with *H. hepaticus*, whereas no such effect was observed in the mice treated with the ΔCdtB strain (Figure 2C). Furthermore, both the protein and mRNA levels of ZO-1 were increased in the ΔCdtB-infected group in comparison to the WT *H. hepaticus*-infected group. Consistently, comparable results were obtained in the intestinal organoid model (Figure S2). These findings indicate that CdtB is a key factor in the disruption of colonic tight junctions by *H. hepaticus*.

### 3.3. CdtB Is Dominant in H. hepaticus-Induced Intestinal Epithelial Cell Damage

**Figure 3:**
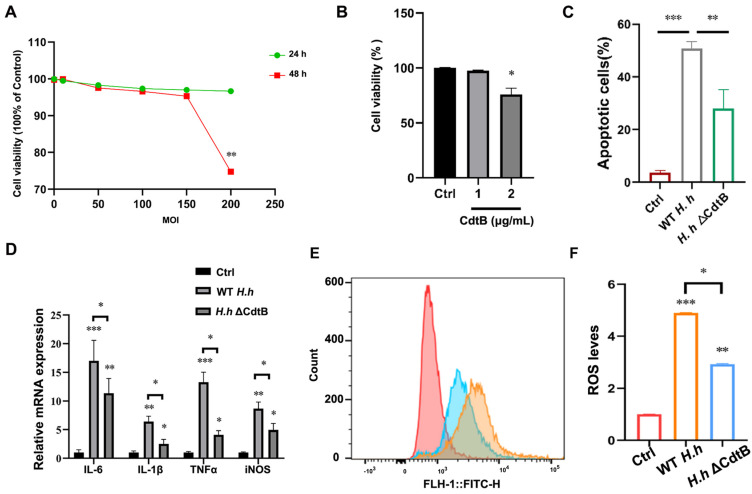
CdtB is attributed to *H. hepaticus*-induced intestinal epithelial cell damage. (**A**) IEC 6 cells were treated with 0–200 MOI *H. hepaticus* for 24 h and 48 h, respectively; (**B**) alternatively, they were treated with different dozens of CdtB protein, prepared in our previous studies, and a CCK-8 assay was performed to evaluated the cell viability. IEC 6 cells were infected with WT *H. hepaticus* or the ΔCdtB strain for 24 h. (**C**) Apoptotic cells were analyzed by using FCM. (**D**) The relative mRNA expression levels of *IL-6*, *IL1β*, *TNF-α*, and *iNOS* were tested by q-PCR. (**E**,**F**) The cellular ROS content was measured and analyzed by using FCM. * *p* < 0.05, ** *p* < 0.01, and *** *p* < 0.001.

**Figure 4:**
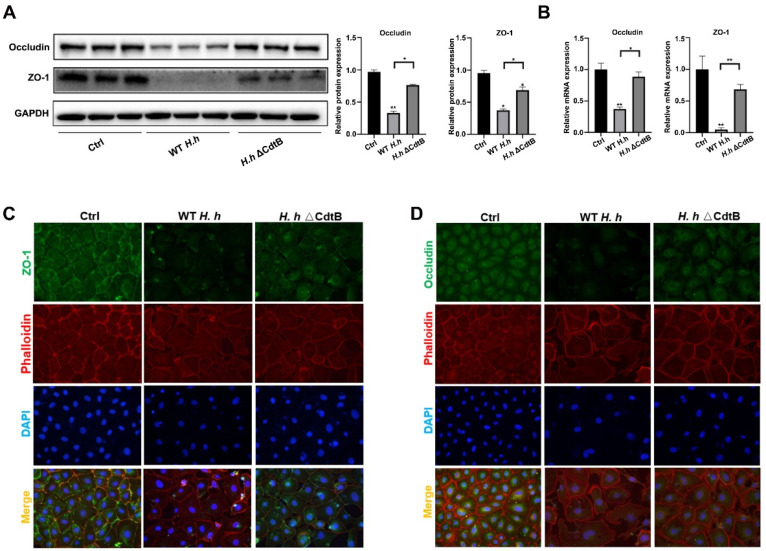
*H. hepaticus* CdtB disturbs the expression and distribution of tight junction proteins in intestinal epithelial cells. IEC 6 cells were treated with WT *H. hepaticus* or the ΔCdtB strain for 24 h. (**A**) Representative occludin and ZO-1 bands from Western blot analyses are shown. Densitometric values were normalized to the GAPDH value. (**B**) The relative mRNA expression levels of occludin and ZO-1 by q-PCR. Representative slides from the control, WT *H. hepaticus*, and *H. hepaticus* ΔCdtB groups stained for (**C**) ZO-1 (green) or (**D**) occludin (green) colocalized with phalloidin (red) were observed using confocal laser scanning microscopy. * *p* < 0.05; ** *p* < 0.01.

To investigate the effects of *H. hepaticus* CdtB on the viability of intestinal epithelial cells, IEC6 cells were treated with different concentrations of *H. hepaticus* or CdtB recombinant protein for 24 h and 48 h. Cell viability was assessed using a CCK-8 assay. As shown in Figure 3A, a significant reduction in cell viability was observed in cells infected with *H. hepaticus* (MOI = 200) for 48 h, while the CdtB recombinant protein (2 μg/mL) demonstrated a notable decline in cell viability at 24 h (Figure 3B). Subsequently, flow cytometry (FCM) was employed to ascertain whether these alterations were linked to CdtB-induced apoptosis. As illustrated in Figure 3C, the CdtB mutation resulted in a reduction in the apoptotic rate of IEC-6 cells relative to the WT *H. hepaticus*-infected group. Moreover, infection with *H. hepaticus* ΔCdtB was found to result in a notable reduction in the mRNA transcription levels of inflammatory cytokines in comparison to those observed in the WT strains. Moreover, comparable effects of CdtB on ROS were discerned in cells utilizing FCM (Figure 4B). These findings suggest that *H. hepaticus* CdtB reduces the viability of intestinal epithelial cells by triggering an inflammatory and oxidative stress response.

### 3.4. CdtB Causes Intestinal Barrier Disruption via MLCK/pMLC2 Signaling Pathway

**Figure 5:**
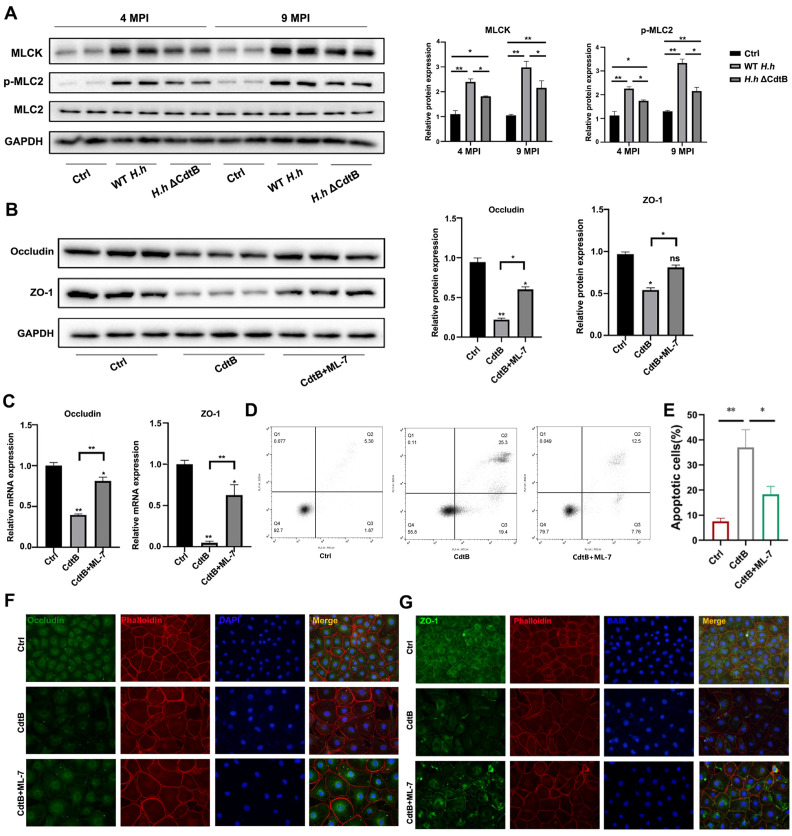
MLCK inhibition suppresses the impairment of tight junctions in intestinal epithelial cells. IEC 6 cells were treated with WT *H. Hepaticus* or the ΔCdtB strain for 24 h. (**A**) Representative MLCK, p-MLC2, and p-MLC2 bands from Western blot analyses are shown. Densitometric values were normalized to the GAPDH value. Cells were treated with CdtB protein in the presence or absence of ML-7. (**B**) Representative occluding and ZO-1 bands from Western blot analyses are shown. (**C**) The relative mRNA expression levels of occluding and ZO-1 obtained by q-PCR. (**D**) Apoptotic cells were analyzed by using FCM. Representative pictures of cells stained for (**E**,**F**) ZO-1 (green) or (**G**) occludin (green) colocalized with phalloidin (red) were observed using confocal laser scanning microscopy. * *p* < 0.05; ** *p* < 0.01. ns: not significant.

To assess the effect of CdtB on tight junctions in vitro, IEC-6 cells were infected with WT *H. hepaticus* or *H. hepaticus* ΔCdtB for 48 h. Both protein expression and mRNA levels of ZO-1 and occludin were decreased by ΔCdtB in cells infected with *H. hepaticus* (Figure 4A,B). Furthermore, exposure to *H. hepaticus* resulted in a marked disruption of ZO-1 distribution, which was impaired by ΔCdtB (Figure 4C). In addition, *H. hepaticus* was detected to significantly reduce the fluorescence intensity of occludin, which was impeded by ΔCdtB as well. It has been demonstrated that MLCK/pMLC2 signaling plays a pivotal role in maintaining the regulation of the intestinal barrier [25]. To ascertain whether MLCK, pMLC2, and MCL2 were involved in the disruption of the intestinal barrier caused by *H. hepaticus* CdtB, their protein expressions were detected by Western blotting. As shown in Figure 5A, the expression of MLCK and the phosphorylation of MCL2 were promoted by *H. hepaticus,* and they were compromised by ΔCdtB (Figure 5A). To further investigate the potential involvement of MLCK/pMLC2 in CdtB-induced intestinal barrier disruption, ML-7, an MLCK inhibitor, was employed to assess the integrity of tight junctions in cells impaired by CdtB. As illustrated in Figure 5B,C, ML-7 was observed to inhibit the reduction in protein and mRNA levels of ZO-1 and occludin, respectively, induced by CdtB. Furthermore, the inhibition of cell apoptosis induced by CdtB was observed with the addition of ML-7 (Figure 5D,E). Furthermore, the redistribution of ZO-1 and occludin was restored by ML-7 (Figure 5F,G). Collectively, these findings suggest that CdtB causes intestinal barrier injury via the MLCK/pMLC2 pathway.

## 4. Discussion

In recent decades, CDTs have been identified as a potential contributor to inflammatory diseases induced by CDT-producing bacteria [26,27,28]. Multiple pathogenic Gram-negative bacteria, including *Aggregatibacter* (formerly *Actinobacillus*) *actinomycetemcomitans*, *Campylobacter* spp., *Escherichia coli*, *Haemophilus ducreyi*, *Helicobacter* species, *Salmonella enterica* serovar Typhi (*S*. Typhi), and *Shigella* species, have been found to produce CDTs, which pose a threat to chickens, rabbits, canines, and humans [29,30,31]. Thus, a suitable model should be established for their exploration. CDTs are essential for the persistent colonization of toxin-producing *H. hepaticus* in mice [32]. In this study, *H. hepaticus* was employed to increase the understanding of the virulent properties of CDTs, which can help in creating prophylactic and therapeutic strategies to combat infectious diseases such as diarrhea in animals and inflammatory bowel disease in humans. The findings of this study demonstrate that *H. hepaticus* infection is associated with the promotion of intestinal inflammation and damage to the intestinal barrier in BALB/c male mice, with CdtB emerging as a key factor in this process. Particularly, CdtB disrupts intercellular tight junction structures and induces apoptosis in IEC-6 cells via the MLCK/pMLC2 signaling pathway.

*H. hepaticus* has been demonstrated to induce experimental colitis in rodents with a fully developed gastrointestinal tract or a developing intestine [33]. In this study, the presence of typical colitis lesions was observed in mice infected with *H. hepaticus*. In particular, the *H. hepaticus* ΔCdtB strain was employed to assess the impact of CdtB on *H. hepaticus*-induced colitis. The restoration of intestinal pathological changes and inflammatory factors induced by *H. hepaticus* by CdtB deletion indicates that CdtB plays a pivotal role in *H. hepaticus*-induced colitis. These observations are in accordance with previous reports on the role of CdtB in *H. hepaticus*-induced colitis [18,34]. The disruption of the intestinal barrier represents a pivotal mechanism in the pathogenesis of intestinal pathogen-induced diarrhea. The function of the cell–cell tight junction in epithelial barrier dysfunction has been documented, yet it has never been examined in the context of *H. hepaticus* infection. Barrier-relevant tight junction proteins, such as ZO-1, occludin, and claudin, are predominantly multiprotein complexes anchored to the cytoskeleton [34,35]. Their impairment causes the leak flux mechanism by increased paracellular permeability [36]. The expression and distribution of tight junction proteins were investigated. To illustrate an intact tight junction network, we demonstrated the colocalization of occludin with ZO-1 in the control samples. CdtB is indispensable for the *H. hepaticus*-induced disruption of barrier-relevant TJ proteins. It is noteworthy that our findings reveal that CdtB induced the redistribution of ZO-1 yet had no effect on occludin. Similarly, no alteration in the distribution of occludin to intracellular compartments was evident in the context of *C. jejuni*-induced disruption of the intestinal epithelial barrier [37]. We propose ongoing changes in tight junction protein expression and distribution with reported effects on barrier function. Further studies are needed to confirm the non-essential role of CdtA or CdtC in virulence by using CdtA or CdtC mutants.

Both inflammation and epithelial cell death are inducing factors of barrier dysfunction induced by bacterial pathogens [37]. Treatment with inflammatory factors was shown to cause barrier dysfunction by tight junction alterations [38]. TNFα-induced barrier dysfunction mostly happens during apoptosis. However, the immune response to the interaction with *C. jejuni* caused barrier impairment and induced overlapping effects of apoptosis and tight junction changes [39]. In terms of inflammatory induction, IL6, TNFα, and IL1β are the major cytokines altered by CdtB mutation during *H. hepaticus* infection. *H. hepaticus* CdtB has been reported to promote IL-6, TNFα, and caspase-3 activity in liver cells [29]. In addition, it was indicated to increase host DNA damage by inducing DNA double-strand break that can upregulate the expression of IL6 and TNFα [18]. Zhu et al. found that CdtB promotes colitis development in IL10^-/-^ mice by the induction of an inflammatory response [40]. In these models, higher levels of pro-inflammatory cytokines were observed in infections with *H. hepaticus*. Our findings consistently demonstrate that CdtB deficiency resulted in a reduction in *H. hepaticus*-induced apoptosis levels in addition to a decrease in pro-inflammatory cytokines. Therefore, it is possible that there are indirect effects of inflammatory factors on *H. hepaticus* CdtB-induced apoptosis, and further investigations in this area would be beneficial.

The cytoskeleton is essential for the regulation of TJ proteins. In this study, we found that CdtB of *H. hepaticus* disrupts the cytoskeleton of the monolayer intestinal barrier in IEC-6 cells. This finding is consistent with a previous study which showed that both Helicobacter pullorum and *H. hepaticus* CdtB induce nuclear and cytoskeletal remodeling [41,42]. Moreover, CdtB raises ROS levels in IEC-6 cell monolayers, which contributes to intestinal inflammation and intestinal barrier disruption [43,44]. MLCK-driven MLC phosphorylation has been found to be involved in signal transduction pathways employed by diverse extracellular stimuli to influence barrier function and alter tight junction protein expression and localization [25,45]. Su et al. found that MLCK^−/−^ mice effectively inhibited apoptosis intestinal epithelial cells and the disruption of TJs caused by DSS [46]. In comparison, it was observed that apoptosis and tight junction disruption were markedly reduced in IEC-6 cells following the administration of an MLCK inhibitor. This finding suggests that ROS-mediated MLCK activation plays a role in the CdtB-induced colonic mucosal barrier [47]. All of these results suggest that ROS and inflammatory cytokines are involved in the activation of MLCK/pMLC2 signaling induced by CdtB, leading to cytoskeletal disruption and the redistribution of TJ proteins. Since CdtB is already known to be essential for virulence, this study primarily extends existing knowledge laterally. Further research is necessary to determine new functions of CdtB and how it contributes to the *H. hepaticus*-induced intestinal pathogenesis.

## 5. Conclusions

In conclusion, *H. hepaticus* CdtB can destabilize and down-regulate tight junction proteins by activating the MLCK-MLC signaling pathway, thus ameliorating the tight junctions of intestinal epithelial cells. CDT plays an important role in *H. hepaticus* infection and the virulence of microbially induced gastrointestinal diseases. Our findings can help in the development of new prevention strategies for intestinal infectious diseases in animals infected by CDT-producing bacteria.

## Data Availability

The data that support the findings of this study are available from the corresponding author upon reasonable request.

## Supplementary Materials

The following supporting information can be downloaded at: https://www.mdpi.com/article/10.3390/vetsci12020174/s1, Figure S1: Intestinal colonization of *H. hepaticus* in the is independent of CdtB; Figure S2. *H. hepaticus* CdtB disturbs the expression and distribution of the tight junction proteins in intestinal organoids; Figure S3. The original images of Figure 1D in the article; Figure S4. The original images of Figure 2D in the article; Figure S5. The original images of Figure 4A in the article; Figure S6. The original images of Figure 5A in the article; Figure S7. The original images of Figure 5B in the article.
