# Supplementary material for: Helicobacter hepaticus CdtB Triggers Colonic Mucosal Barrier Disruption in Mice via Epithelial Tight Junction Impairment Mediated by MLCK/pMLC2 Signaling Pathway
Source: Vet Sci. 2025 Feb 14;12(2):174. doi: 10.3390/vetsci12020174 (PMC11860670; doi:10.3390/vetsci12020174)
Supplement: Supplementary file 1 [file vetsci-12-00174-s001.zip › vetsci-3428211-supplementary.pdf]

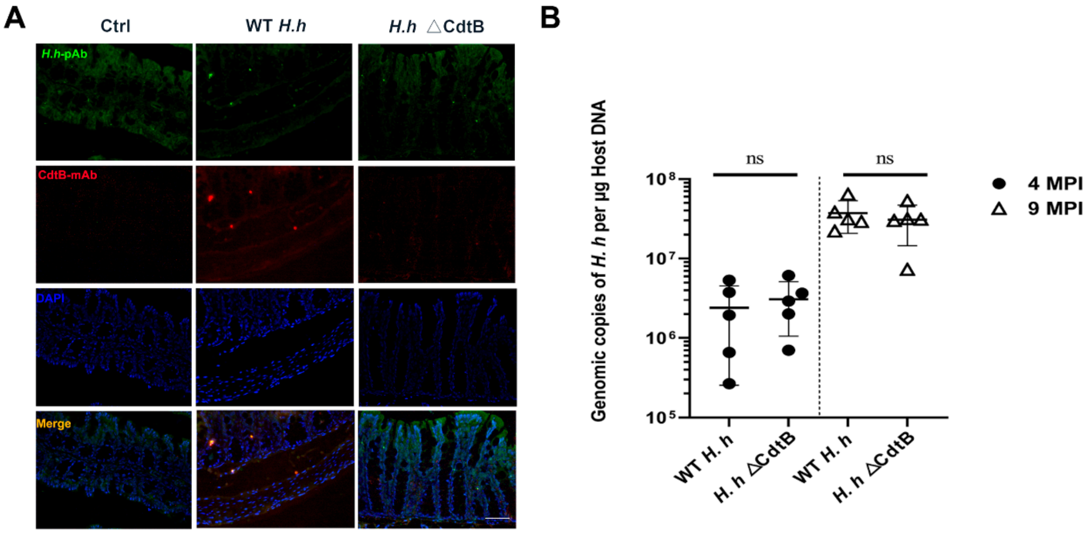

Figure S1. Intestinal colonization of *H. hepaticus* in the is independent of CdtB. Mice were treated with WT *H. hepaticus* or ΔCdtB strain by gavage for 4 or 9 months. (A) Representative pictures of intestinal tissues labeled for *H. hepaticus* (green) and CdtB (red) were observed using confocal laser scanning microscopy. (B) Genomic copies of *H. hepaticus* in the intestinal tissues of mice infected with WT *H. hepaticus* or ΔCdtB strain was analyzed by q-PCR.

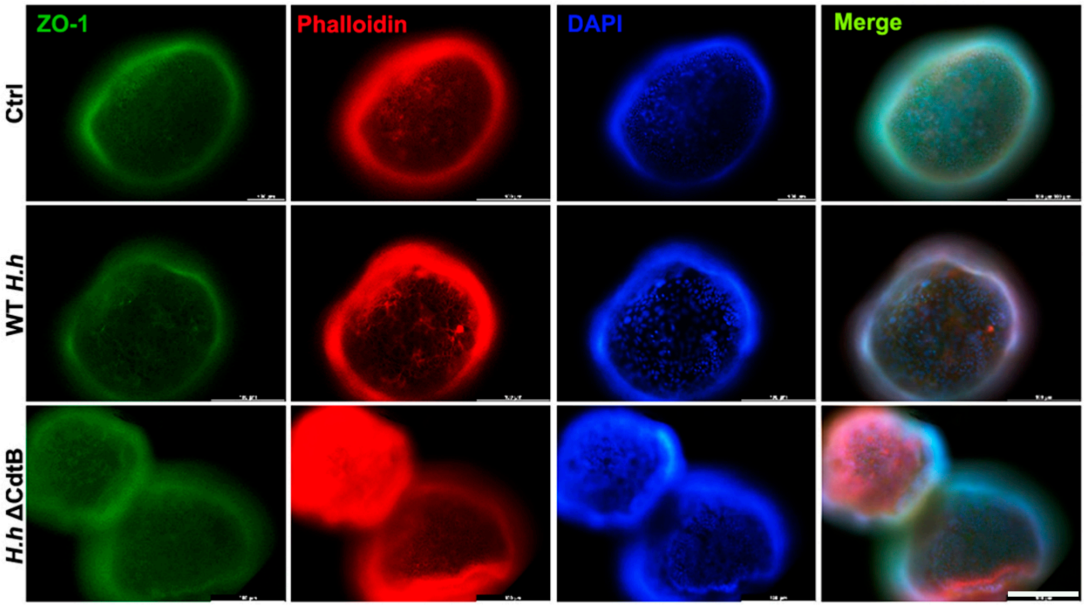

Figure S2. *H. hepaticus* CdtB disturbs the expression and distribution of the tight junction proteins in intestinal organoids. Intestinal organoids were infected with WT *H. hepaticus* or ΔCdtB strain for 24 h. Representative pictures of cells stained for ZO-1 (green) or phalloidin (red) were observed using confocal laser scanning microscopy. Bar=100 µm, * $p<0.05$.

Original images

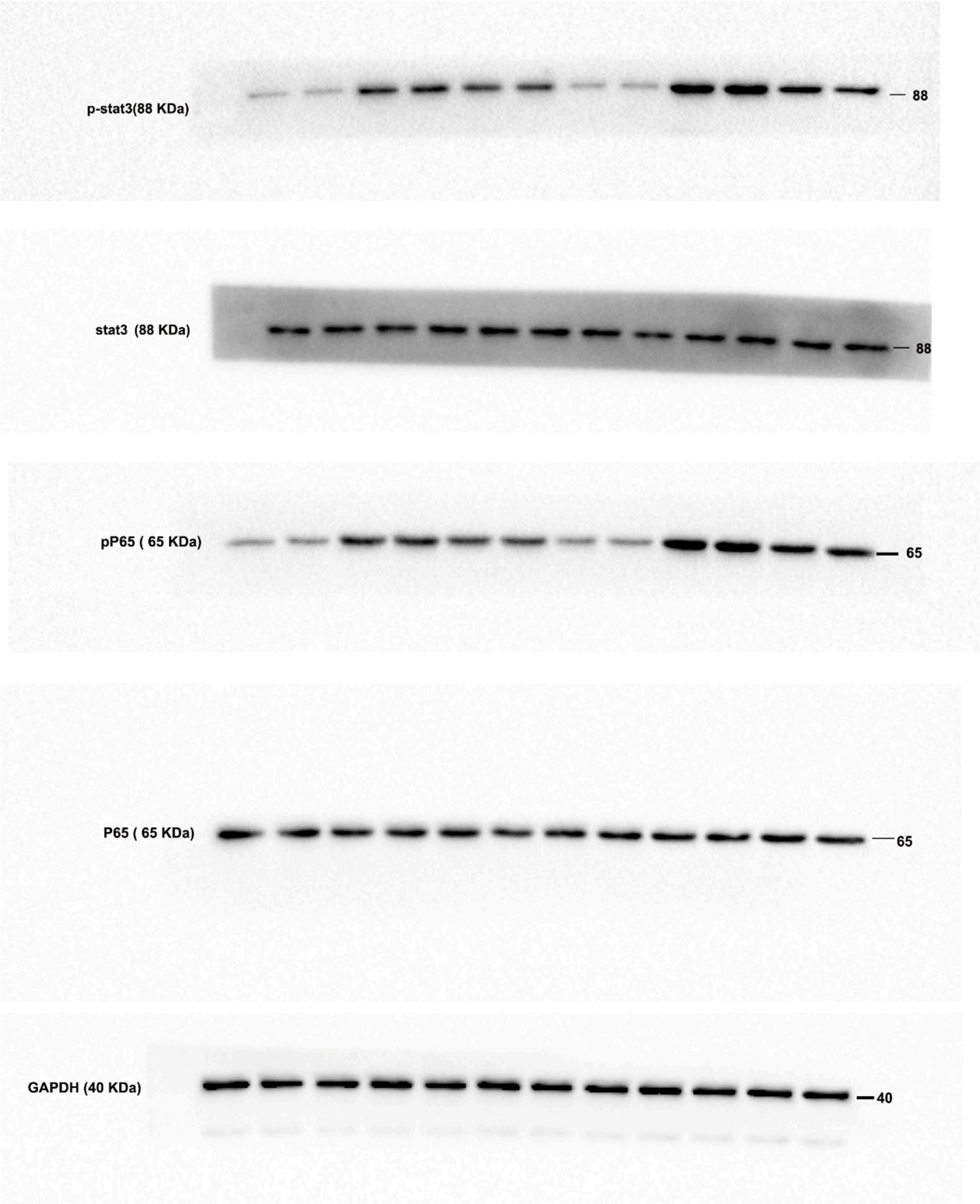

Figure S3. The original images of Figure 1D in the article

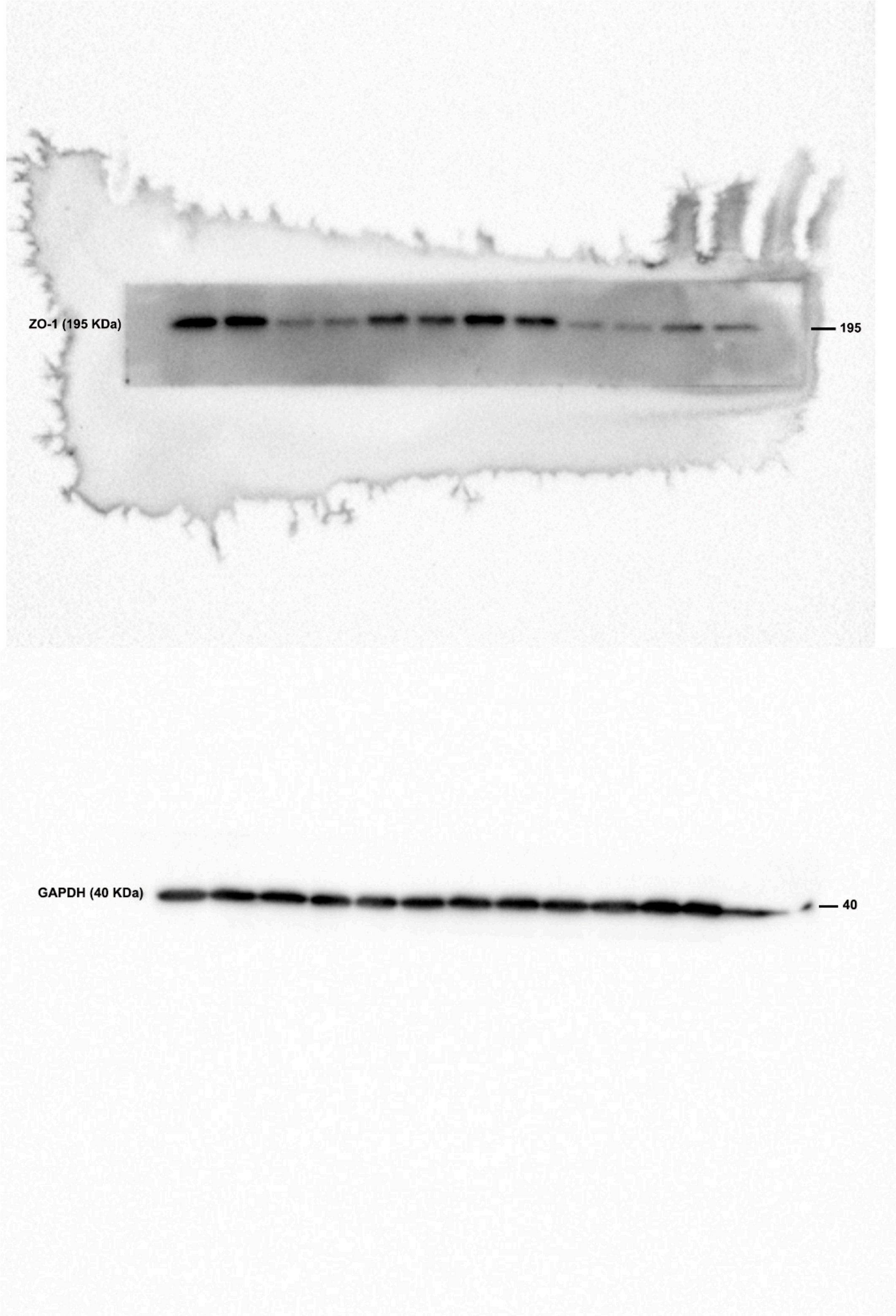

Figure S4. The original images of Figure 2D in the article

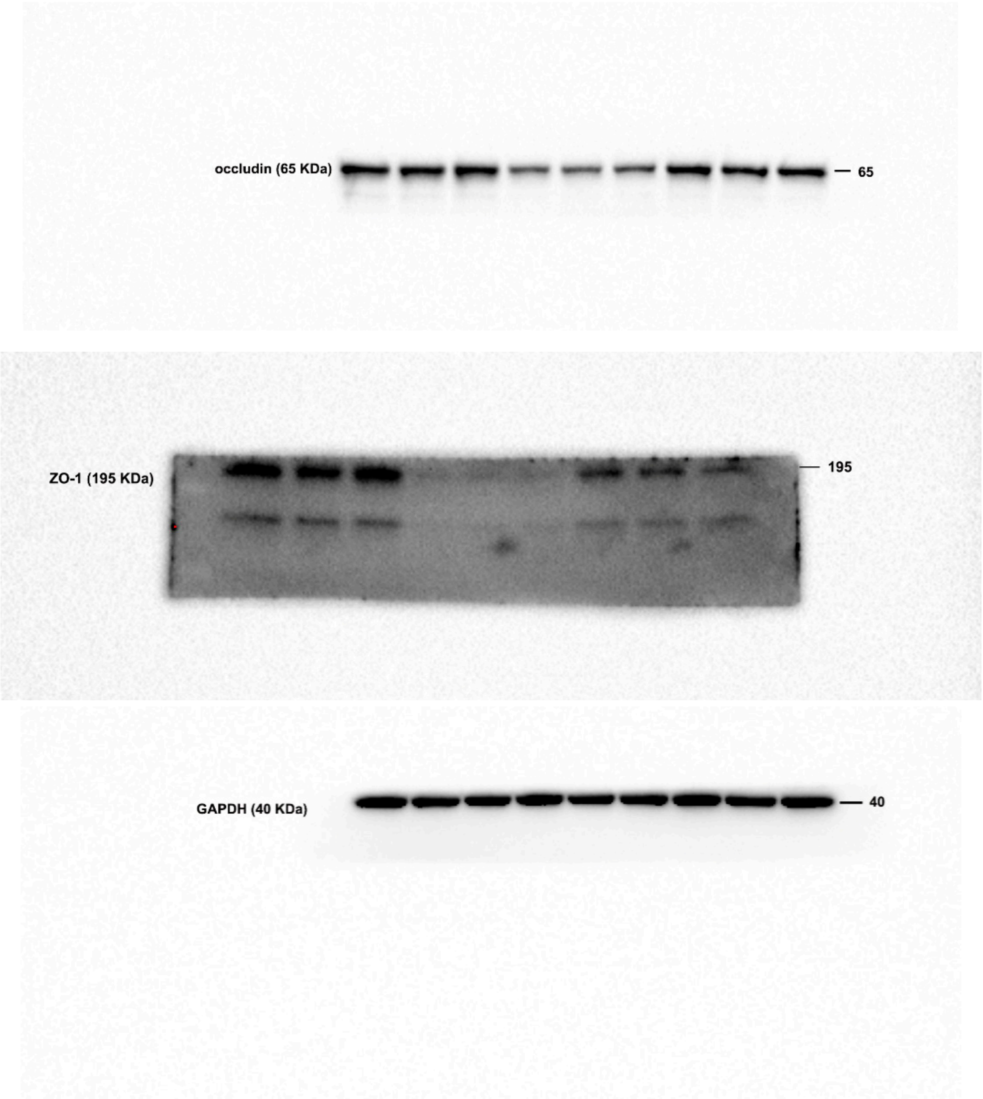

Figure S5. The original images of Figure 4A in the article

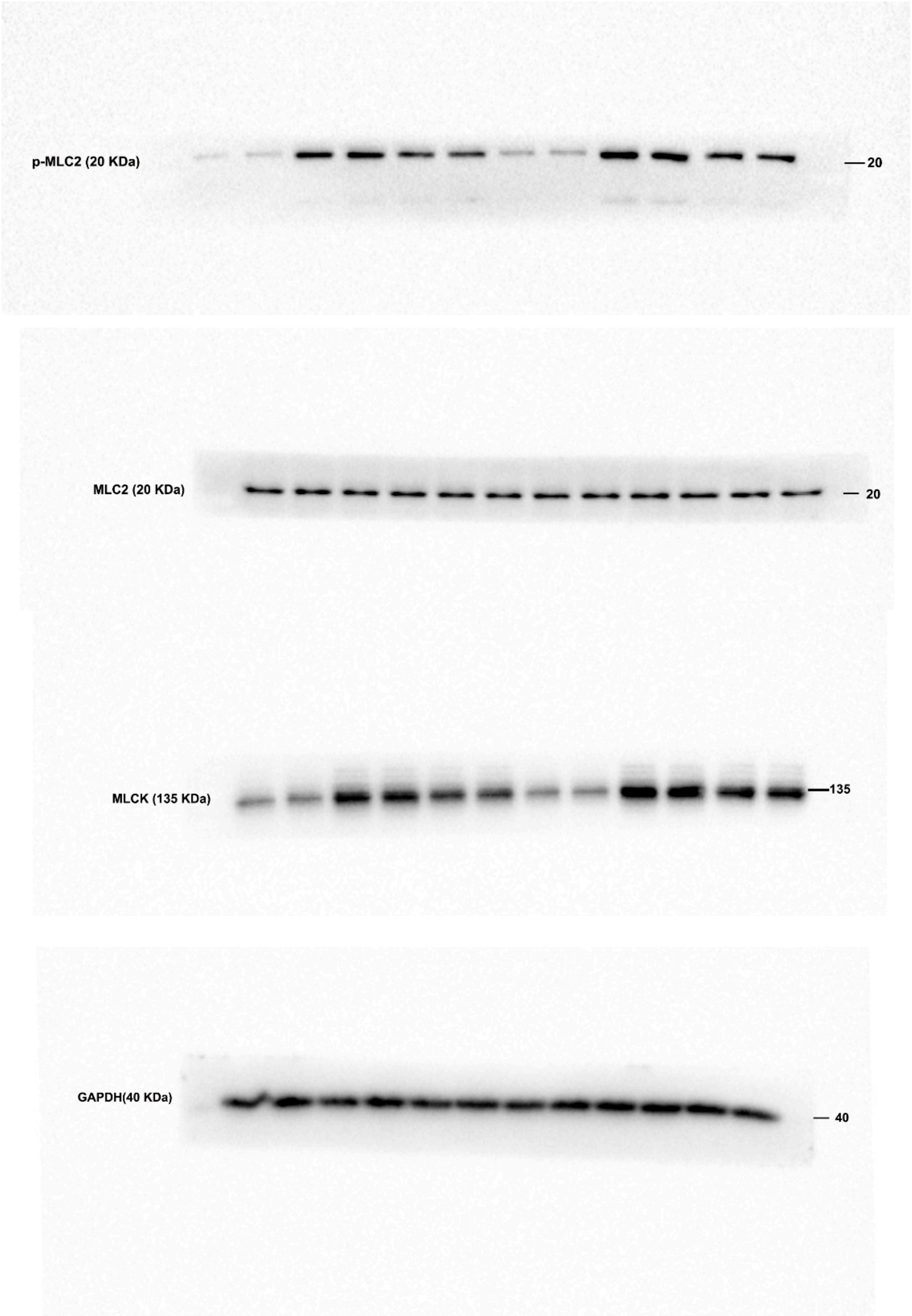

Figure S6. The original images of Figure 5A in the article

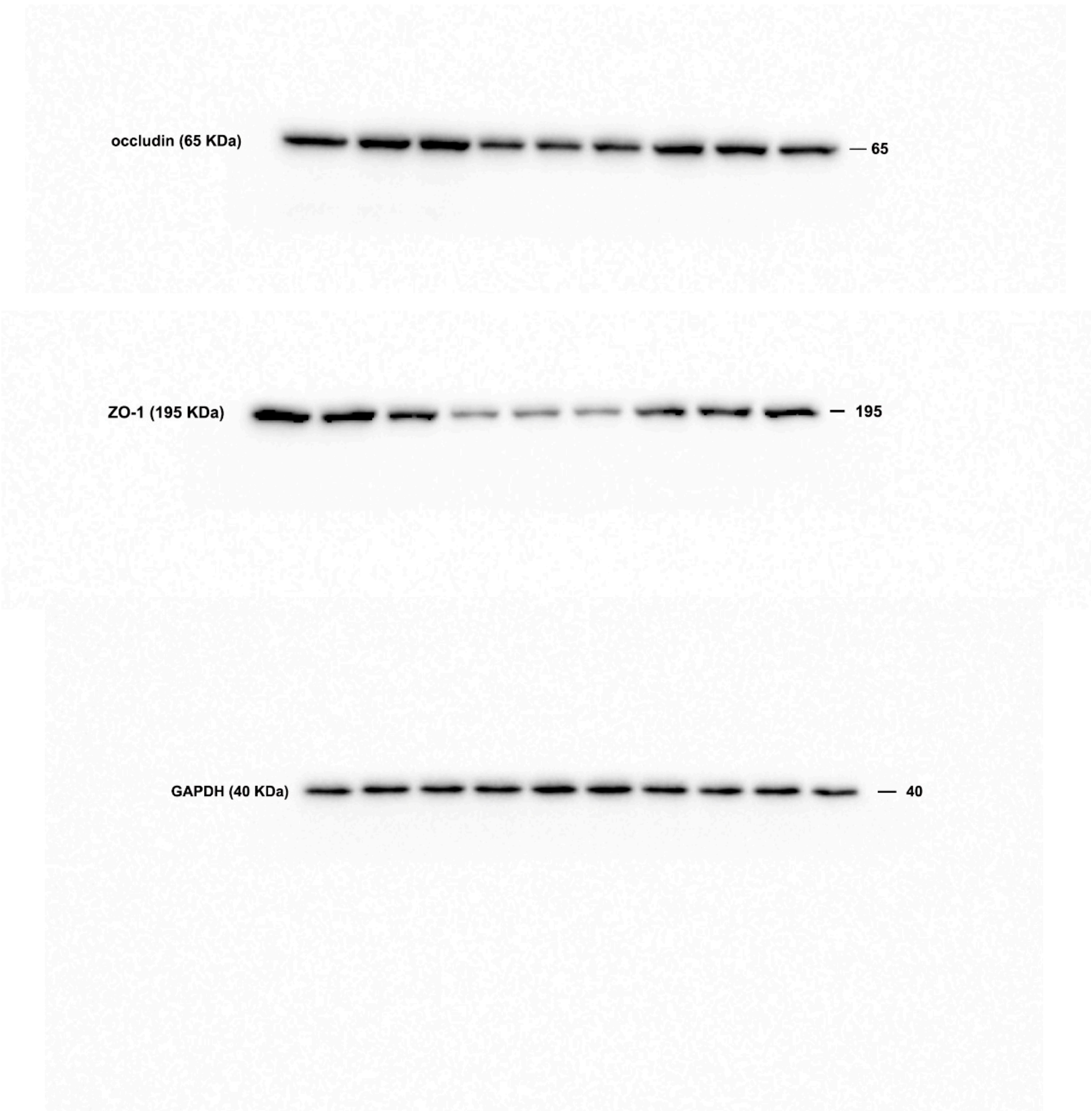

Figure S7. The original images of Figure 5B in the article
